# The Development and Application Evaluation of Meridian Energy Detection System in Traditional Oriental Medicine: A Preliminary Study

**DOI:** 10.1155/2018/9469703

**Published:** 2018-08-06

**Authors:** Yu-Chen Lee, Hui Ping Ng, Yung-Hsien Chang, Wen-Chao Ho

**Affiliations:** ^1^Graduate Institute of Acupuncture Science, China Medical University, Taichung, Taiwan; ^2^Acupuncture Department, China Medical University Hospital, Taichung, Taiwan; ^3^International Master Program in Acupuncture, China Medical University, Taichung, Taiwan; ^4^Department of Public Health, China Medical University, Taichung, Taiwan

## Abstract

As technology advances, more modern medical devices are developed to help the physicians in performing objective assessment and diagnosis. In this study, our main objective was to evaluate the clinical application of the low voltage Meridian Energy Detection System in assessing the electrodermal activity (EDA) of the specific acupoints in a specific age group of healthy individuals and to assess the difference in the energy overview between the genders and specific time of assessment. 43 young healthy adults were recruited in a single group, nonrandomized, evaluation study. Written informed consent of each participant was obtained prior to the assessments. Results on energy overview between genders and specific time of assessment as well as factors influencing EDA were discussed. It was concluded that the study using Meridian Energy Detection System in healthy individuals provided an understanding of the difference in energy level of the meridians between the genders. Male healthy individuals had significantly higher values for Physical Status as well as Yin and Yang energy while female healthy individuals had significantly higher values for Mental Health and Autonomic Nervous System. There was no significant difference when comparing the assessments at the specific time of assessment. Hence there was no specific time in using the device. However, due to the limitation of the sample size and the healthy subjects, future research can be designed to investigate whether the time of assessment can affect the results in individuals with specific disease conditions in larger scale. It may merit further studies on the application of such device as preliminary diagnosis of the overall conditions and investigate the treatment efficacy by observing the change in the meridian energy level.

## 1. Introduction

As technology advances, more modern medical devices are developed to help the physicians in performing objective assessment and diagnosis. It was a step of advancement in clinical practice of Traditional Oriental Medicine since 1950 when devices for pulse diagnosis and meridian energy analysis were first developed. Experimental and translational studies are required for evaluating the clinical application of the modern tools in Traditional Oriental Medicine.

Based on the ancient publication “Yellow Emperor's Inner Canon” (Huang Di Nei Jing) compiled in the period of the Warring States (475-221 BC), the meridian system in the human body consists of twelve primary pathways (also known as “Primary Meridians” or “Channels and collaterals”). In the book of ‘Magic Pivot, Chapter 33-Discourse on the Seas', the original text is translated as ‘The twelve Primary Meridians in the body penetrate the interior visceral and bowels (also termed as ‘*zang-fu' or organs*) and connect the exterior limbs and joints'. In the book of ‘Magic Pivot, Chapter 47-The Visceral', the original text is translated as ‘The meridians are the pathways of the Qi (vital energy) and blood, nutrify the Yin and Yang, nourish the tendons and bones and lubricate the joint' [1, 2].

**Figure 1:**
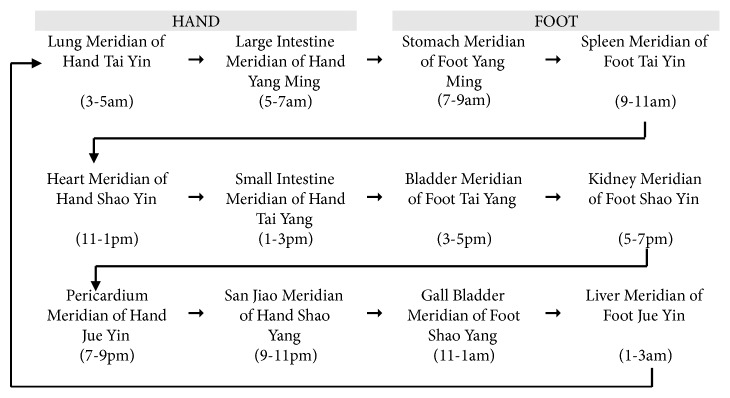
Meridian flow chart.

Based on the TCM theory, the meridians are longitudinally and laterally interconnected pathways that are distributed throughout the body to provide nourishment to all the body tissues and organs. The twelve Primary Meridians and eight Extraordinary Meridians play important roles in the normal physiological functions of the body. The movement of Primary Meridian Qi is usually in a specific direction as illustrated in Figure 1 [2, 3].

The acupoints (or acupuncture points) are specific anatomical defined areas on the body along the meridians where Qi is gathered. These points are usually used for application of acupuncture in treatment of diseases.

Each of the twelve Primary Meridians has specific important group of acupoints on the upper and lower limbs, located distal to the elbows and knees. They are the ‘Five Shu-points', also known as “Five Transport points”.  Table 1 describes the characteristics of the Qi flow in these acupoints [2, 3].

The Yuan-Source points were first discussed in the book of ‘Magic Pivot, Chapter 1-The Nine Needles and Twelve Source'. The original text is translated as “The five* zang *(visceral) has six* fu *(bowels). Six* fu* have twelve Yuan-Source points which locate at the 4 joints of the extremities (wrists and ankles) that can treat the five* zang*. When five* zang* are diseased, the Yuan-Source points should be selected [1].” There are total 24 Yuan-Source points on both hands and feet.

In the 1950s, the electrical detection of acupoints was firstly introduced by various researchers, namely, Reinhard Voll (1953, Germany) [4], Yoshio Natakani (1956, Japan), and J.E.H. Niboyet (1957, France). Identifiable electrical characteristics of the skin points and the resembled traditional acupoints were independently concluded [5].

**Figure 2:**
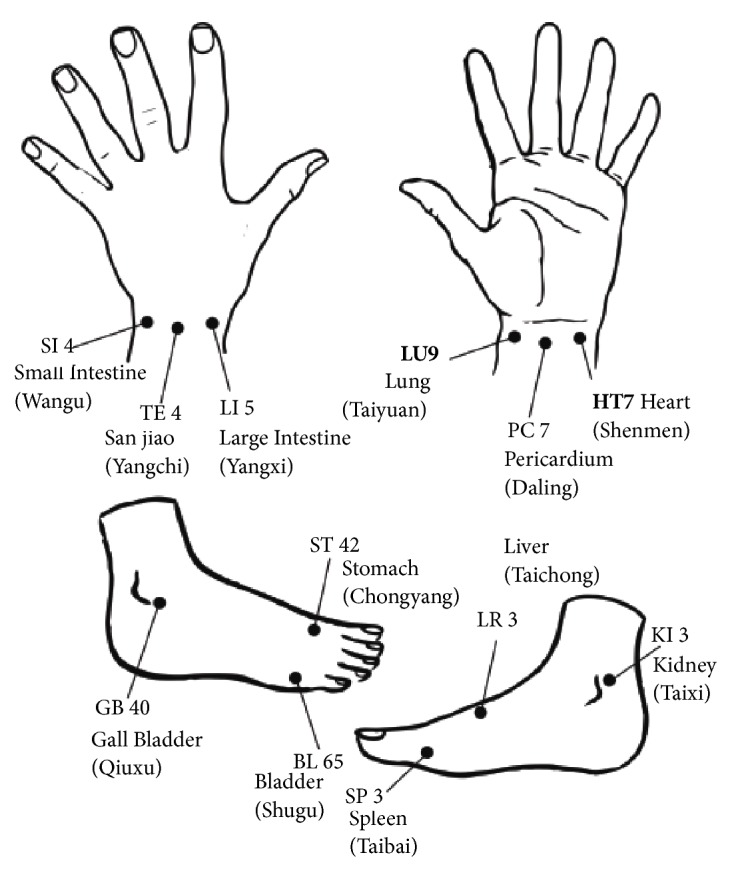
Representative measuring points.

Dr. Yoshio Nakatani discovered a series of high electrical conductivity points that run longitudinally on the body which matched closely to the meridian acupoints [6]. He called these lines with high electrical conductivity points as Ryodoraku (Ryo=good; do=electrical conductivity; raku=line) while the points were called Ryodoten. Coincidentally, majority of these most energetically active points on the meridians corresponded to the Yuan-Source points that were located at the wrists and ankles. These were referred to as Representative Measuring Points (RMP) as illustrated in Table 2 and Figure 2. In his study, an amperometer (12V, 200 uA) was used to assess these points. Instead of absolute reading, a normalized scale of 0-200uA was reported due to the high variability in skin conductance measurements [4]. It was believed that an increase in conductance (i.e., decreased resistance) represents a surfeit of energy in the respective meridian while a decrease in conductance (i.e., increased resistance) represents a deficiency of energy in the respective meridian [7].

In fact, the Ryodoraku had incorporated the concept of electrodermal conductivity in the system. Electrodermal activity (EDA) was first introduced in 1966 as a common term to describe the electrical phenomena on the body skin. It is defined as all active and passive electrical characteristics in the skin and the appendages [8]. EDA is the current standardized preferred term for electrodermal response (EDR), psychogalvanic reflex (PGR), galvanic skin response (GSR), skin conductance, skin conductance level (SCL), skin conductance response (SCR), and sympathetic skin response (SSR) [9]. Overlying sweat glands and epidermis are involved in generating the EDA. It is mediated by dorsal thalamus, orbitofrontal cortex, posterior hypothalamus, and ventrolateral reticular formation. The spontaneous response is also known as peripheral autonomic surface potential or sympathetic skin response [10].

Colbert et al. (2008) reported that lower electrical skin resistance and higher capacitance could be found in acupoints compared to the tissues surrounding them; certain clinical diseases might be correlated with higher or lower resistance at specific acupoints; physiologic dysfunction that was experimentally induced and its subsequent recovery had correlated with the changes in electrical impedance at relevant acupoints. Hence electrical skin impedance of acupoints was a unique feature distinct from nonacupoints. Changes in skin impedance at the acupoints might have significant value in the areas of therapeutic, diagnostic, and research [11].

In this study, our main objective is to evaluate the application of low voltage Meridian Energy Detection System in assessing the EDA of the 24 RMP bilaterally on both wrists and ankles in a specific age group of healthy individuals. The mean value of the meridian energy which is represented by the EDA at 2 specific times of measurement has been obtained. The secondary objective is to evaluate the clinical application of the result interpretation between the genders and the specific time of assessment.

To better understand the evaluation, we have streamlined and focused our study on a specific group of healthy individuals aged 20-30 in Taichung city, Taiwan.

## 2. Materials and Methods

### 2.1. Type of Study

A single group, nonrandomized, evaluation study was conducted. Method of randomization and blinding was not considered.

### 2.2. Location of Study

The study was conducted in China Medical University, Taichung, Taiwan. The study protocol was approved under ID: CMUH104-REC1-130 by China Medical University & Hospital Research Ethics Committee.

### 2.3. Subjects

A total of 43 young healthy adult participants were recruited. Written informed consent of each participant was obtained prior to the assessments.

*Eligibility Criteria*. Healthy participants aged 20-30 years who provided signed written informed consent were included.

The participants who met following criteria were excluded in the study:

- Pregnancy or lactation
- Severe diseases such as carcinomas under chemotherapy, psychological/psychiatric disorders, and chronic heart failure
- Have received pacemaker or coronary intravascular stent placement
- Unable to undergo evaluation with the Meridian Energy Detection System
- Alcohol abuse or drug addiction
- Communication disorder
- Refusal to provide informed consent in the study
- Exclusion at Project Investigator's discretion
- Participation in other clinical trials within 3 months

Participants were advised to avoid coffee, tea, or caffeinated beverages before the procedure.

### 2.4. Materials

**Figure 3:**
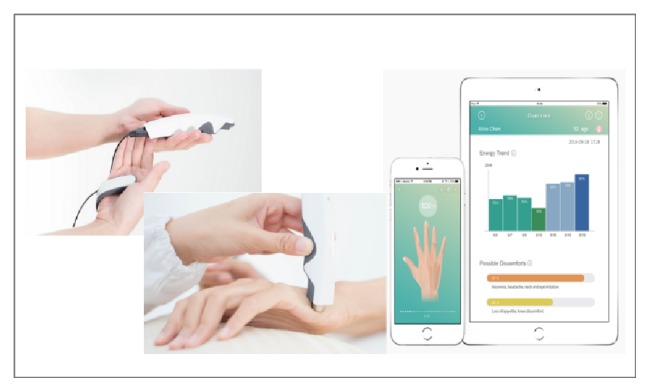
Meridian Energy Detection System.

Aetoscan Meridian Energy Detection System (Aeto Technology Co. Ltd., Taiwan) was used in this study. It was a simple, noninvasive Meridian Energy Detection System (MEDS) that use low voltage electrical current (3.7V, 200 uA) to detect the energy level (or the electrical conductivity) of the 24 RMPs on the skin of wrists and ankles. The device was connected to its mobile application with iOS system support through Bluetooth with connection encryption. The mobile application provided instant results and analysis as shown in Figure 3 [14].

The software mobile application version 1.0.0.0 was installed in iPad. The raw data of assessment was extracted into Microsoft Excel through the web application.

The result interpretation included the following 5 major indices illustrated in Table 3.

### 2.5. Procedure

**Figure 4:**
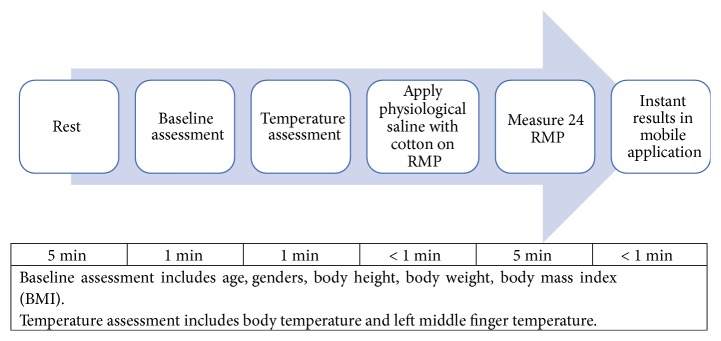
Procedure and duration of assessment.

The following procedure was carried out:

- The assessment was conducted at 2 specific times of the day: 9-11 am (Spleen meridian) and 1-3 pm (Small Intestine meridian).
- Each participant was invited to sit in a room with controlled ambient temperature of 23.1°C.
- For each session, the probe was sterilized with alcohol swab and the device was digitally calibrated to avoid any confounding factors.
- The following procedure was repeated in both sessions as illustrated in Figure 4.
- The participant was asked to grip the U-shape buckle (electrode) on one palm with constant pressure while the investigator placed the probe at each of the 24 RMP perpendicularly to the skin with even pressure without touching the participant's hands or feet.
- During the procedure, the participant was advised to remove any metallic or electronic accessories including watches to avoid electrical disturbance. Electronic devices such as mobile phones were avoided to be used by the participants too.

### 2.6. Statistical Analysis

We calculated the mean and standard deviation for each variable to show the distribution and perform as the descriptive analysis. Further T-test was conducted for the comparison analysis. A two-tailed P-value of 0.05 was considered as significant. SAS statistical software (version 9.4; SAS Institute, Cary, NC) was used to conduct the analyses.

## 3. Results

**Figure 5:**
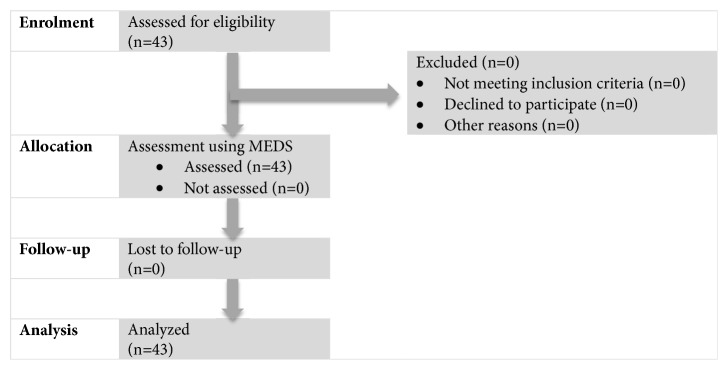
Overview of the study.

A total of 43, including 13 males and 30 females, young healthy adult volunteers were participated in this study. The average age was 26.15±6.97 years and 23.27±2.94 years, respectively. There was no participant dropped out due to discomfort during the procedure or repeated assessments.  Figure 5 illustrates the overview of the study.

### 3.1. Baseline Assessment

The baseline assessment such as body weight and height, body temperature (temporal), middle finger temperature, and body mass index (BMI) were obtained.  Table 4 provides the results of the baseline assessment.

Table 4 concluded that there was significant difference in the body height, body weight, and finger temperature between the genders within the participants.

### 3.2. Energy Overview Interpretation

#### 3.2.1. Energy Overview between the Genders

**Figure 6:**
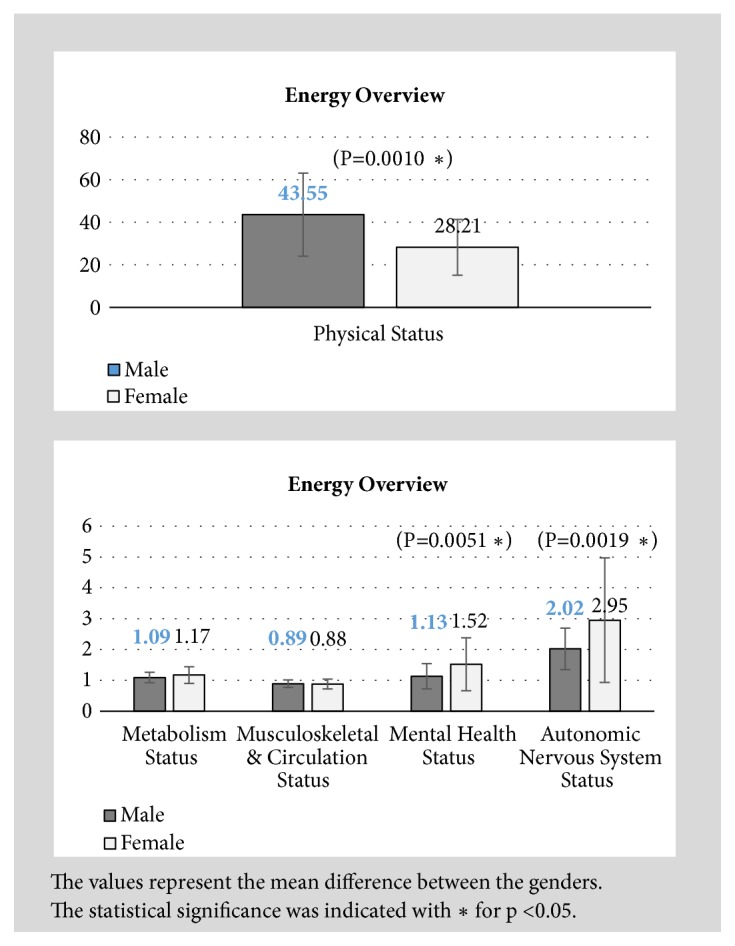
Energy overview between the genders.

Figure 6 provides the comparison of the 5 major indices between the genders.

It was concluded that there were significant differences in the Physical Status, Mental Health Status, and the Autonomic Nervous System status between the genders within the participants. Healthy male participants generally had higher Qi energy level than female. Healthy female participants had higher values for Mental Health and Autonomic Nervous System status than male. Both male and female had overactive Autonomic Nervous System status.

#### 3.2.2. Energy Overview at Specific Time

**Figure 7:**
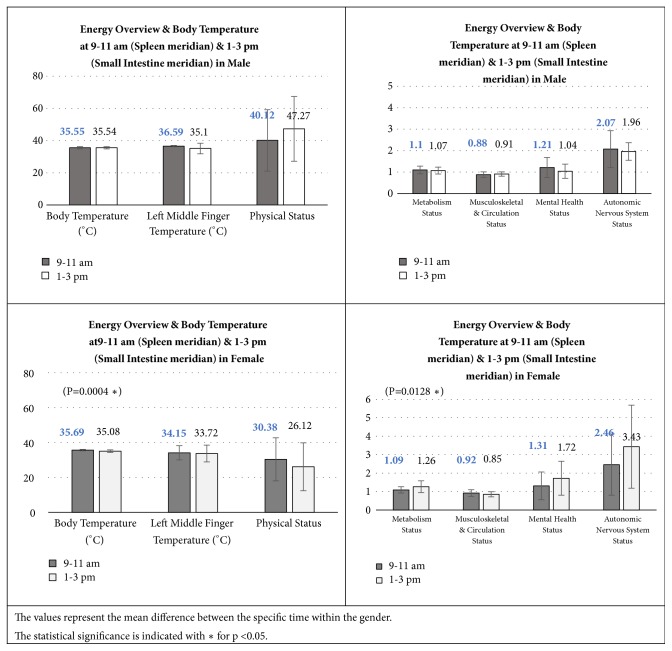
Energy overview at specific time within the genders.

Figure 7 illustrates the comparison of the 5 major indices at specific time within the genders.

It was concluded that there was no significant difference between the time of assessment for male participants but there was significant difference in the body temperature and mean Metabolism Status between the time of assessments for female participants.

#### 3.2.3. Meridian Energy Balance of the Yin and Yang Meridians

**Figure 8:**
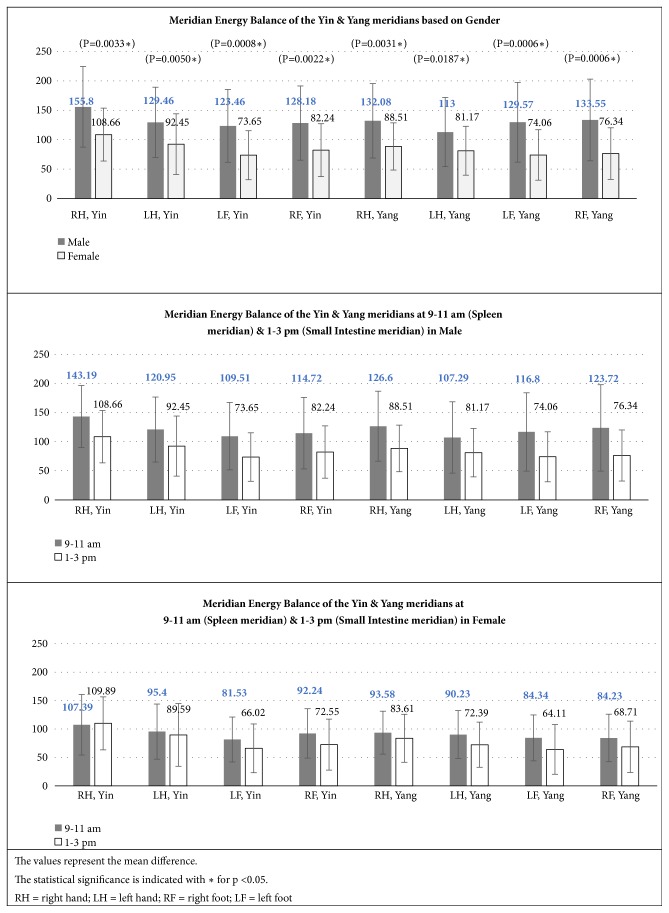
Meridian Energy Balance of the Yin and Yang meridians.

The results were also categorized based on the Yin and Yang meridians of the left/right and hand/foot by summing the values of Physical Status obtained from genders and specific time as illustrated in Figure 8.

It was concluded that the Meridian Energy Balance of the Yin and Yang meridians of female participants was significantly lower than male participants.

There was no significant difference in the Meridian Energy Balance of the Yin and Yang meridians at different time of assessment.

#### 3.2.4. Meridian Energy Balance in Left and Right Meridians

**Figure 9:**
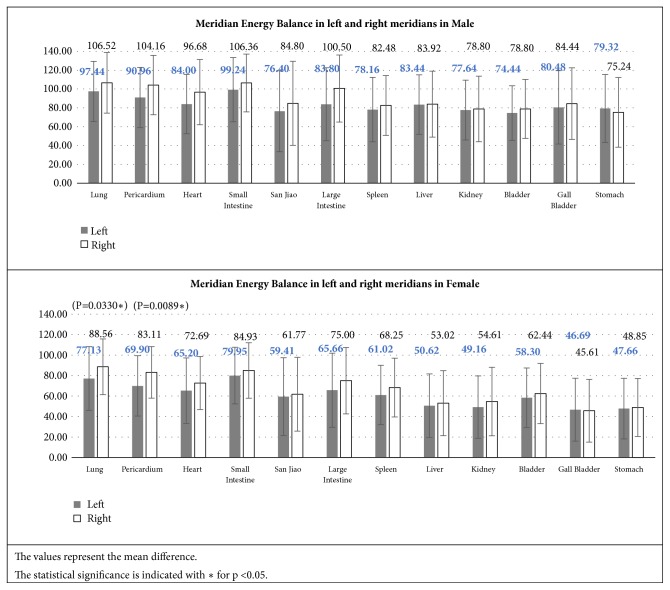
Meridian Energy Balance of the Yin and Yang meridians.

We further categorized and compare the difference in Meridian Energy Balance in the left and right meridians as illustrated in Figure 9.

It was concluded that, in both genders, there was no significant difference in Meridian Energy Balance between the left and right meridians, though the right meridian acupoints have higher skin conductivity than the left with the exception of Lung and Pericardium Meridians in female.

#### 3.2.5. Meridian Energy Balance in Left and Right Meridians at Specific Time

**Figure 10:**
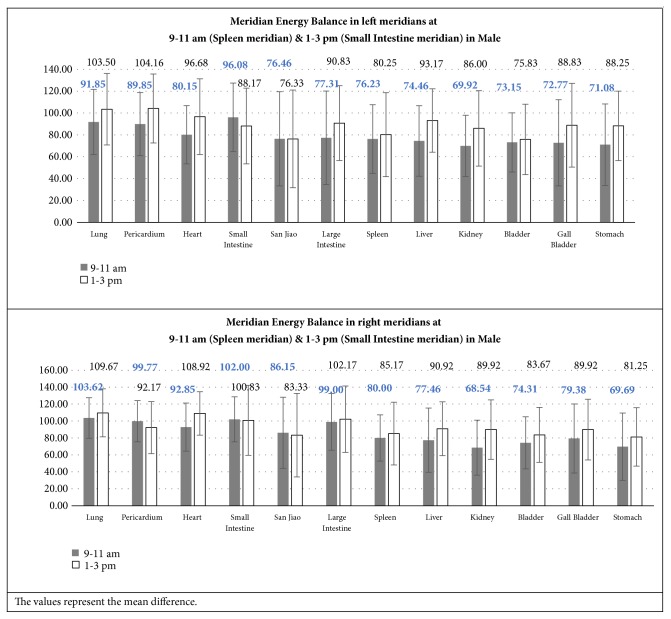
Meridian Energy Balance in left and right meridians at 9-11 am (Spleen meridian) and 1-3 pm (Small Intestine meridian) in Male.

**Figure 11:**
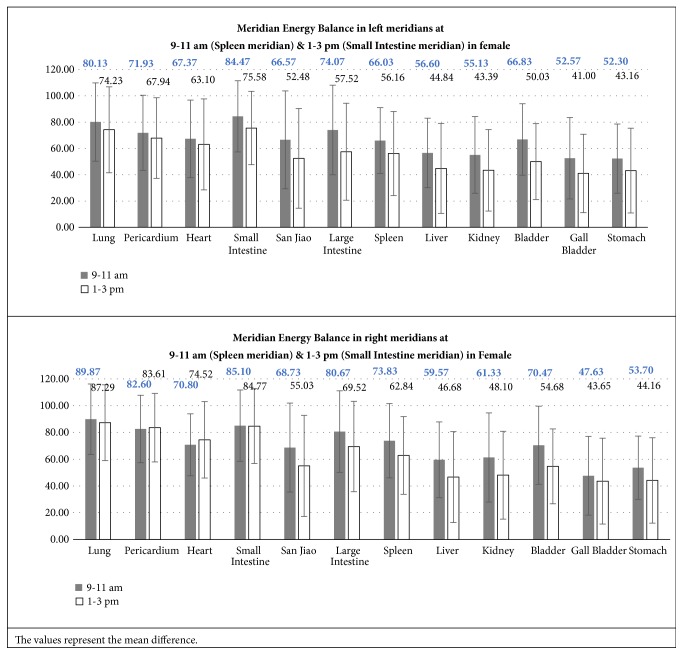
Meridian Energy Balance in left and right meridians at 9-11 am (Spleen meridian) and 1-3 pm (Small Intestine meridian) in Female.

Figures 10 and 11 illustrated the comparison in the left and right meridians during the specific time of assessment.

It was concluded that there was no significant difference between the left and right meridians between the 2 specific times of assessment.

## 4. Discussion

Our specific goal of this clinical study is to evaluate the clinical application of the low voltage Meridian Energy Detection System in assessing the EDA of the 24 RMP bilaterally on both wrists and ankles in a specific age group of healthy individuals and to determine the difference in the energy overview between the genders and specific time of assessment.

### 4.1. Factors That Can Influence Electrodermal Activity

Various technical factors that could influence the electrodermal activity (EDA) on the skin has been reported by Andrew et al. (2007) [5]. These included the skin structures such as the integrity, hydration, and thickness; sweat gland density; electrode polarization such as the electrode material and size, current amplitude, and frequency as well as the contact medium used. Other influential factors discussed in other studies included the contact time on the skin, amount of pressure on the skin, precise location of acupoints, and control environment such as the room temperature as well as the degree of skin moisture. Variability in the measurement has resulted in doubt in the reliability of the measurement of EDA. Experienced operators also played an important role in avoiding any confounding factors and to ensure consistency by having sufficient knowledge regarding the use of the device [15–17].

As EDA can also be influenced by the emotion [18], a controlled ambient environment with constant temperature is maintained in the study. In the study, it was observed that generally, healthy female participants had significantly lower mean finger skin temperature than healthy male participants in the same controlled environment. This was similar to the findings by Kim et al. (1998) that the mean finger temperature of women is about 2.8 degrees lower than men [19]. However due to the small sample size, there was no significant difference in the mean core body temperature between the genders.

Beside the environmental factors, consumption of caffeinated beverages can also affect the electrical conductance. A study by Tsai et al. (2014) reported that the mean values of electrical conductance increased in most of the meridians 30 minutes after coffee consumption. Hence one should avoid caffeinated beverages before using the device [20].

### 4.2. Energy Overview between the Genders

Generally, male participants had significantly higher value in Physical Status than female participants. The Physical Status indicated the average energy balance of all the meridians. The higher the value is, the higher the Qi energy is. This results coincided with the study from S. Chamberlin* et al*. (2011) [21] in which a large scale clinical trial was conducted to determine the influence of age, genders, and time of the day on the skin conductance at the 24 Source points. It was reported that the mean skin conductance at acupoints was higher in male.

It was also observed that healthy female participants had higher values for Mental Health and Autonomic Nervous System status which could be possibly due to the fact that female usually experiences more stress and anxiety easier than male. Both male and female have overactive Autonomic Nervous System status. In studies related to stress and depression among university students, it was reported that female students had higher level of stress, depression, frustration, and anxiety than male students [22, 23].

There was no significant difference between the Metabolism Status and Musculoskeletal and Circulation Status in the genders. This could be due to the fact that the subjects recruited were healthy individuals. Future research may be merited to investigate these indices in unhealthy individuals.

When comparing the Yin and Yang meridian energy, female participants had significantly lower energy level compared to male participants. This affirmed the observation on the Physical Status between the genders that female Qi is more deficient than male.

### 4.3. Energy Overview at Specific Time of Assessment

There was no significant difference in using the device at the specific time of assessment, i.e., 9-11 am and 1-3 pm for Physical Status, Metabolism Status, Musculoskeletal and Circulation Status, and Mental Health and Autonomic Nervous System status except the Metabolism Status in female. This could be due to some exception that required further investigation.

There was no significant difference when comparing the Yin and Yang or left and right meridians at the specific time of assessment.

Hence the device could be used at these two specific periods of the day without causing variation in the results. However, future research can be designed to investigate whether the time of assessment can affect the results in unhealthy individuals.

### 4.4. Limitation in This Study

The limitation in this study is the low sample size. Further study with larger sample size, different age groups, and individuals with various disease conditions to be more conclusive in the clinical application of the device is recommended. Studies on the effect of meridian energy level in specific disease conditions and treatments using Meridian Energy Analysis Device, e.g., in abnormal gastroscopy [24], Low Back Pain [25], and Endometriosis-Related Chronic Pelvic Pain [26], have been reported. Similar studies on other disease conditions can be designed to investigate if such device can be used as preliminary diagnosis as well as for treatment evaluation.

We have excluded the study of other functions in the application that can assist in preliminary diagnosis such as Possible Discomfort, Meridian Summary, and Body System Report. Further research could be done to conclude the accuracy.

## 5. Conclusions

In conclusion, the study using Meridian Energy Detection System in healthy individuals provided an understanding of the difference in energy level of the meridians between the genders. Male healthy individuals had significantly higher values for Physical Status as well as Yin and Yang energy compared to female healthy individuals. Female healthy individuals had significantly higher values for Mental Health and Autonomic Nervous System due to the fact that female experienced stress, depression, and anxiety easier than male. There was no significant difference when comparing the Yin and Yang or left and right meridians at the specific time of assessment. Hence there was no specific time in using the device. However, due to the limitation of the sample size and the healthy subjects, future research can be designed to investigate whether the time of assessment can affect the results in individuals with specific disease conditions in larger scale.

As people are getting more health conscious, the healthcare professionals play an important role in counselling the patients in their health maintenance. Such Meridian Energy Detection System may be used as a tool for preliminary diagnosis of the overall conditions of the individual. It may be used as a tool to investigate the treatment efficacy by observing the change in the meridian energy level. It merited further studies to be more conclusive.

## Figures and Tables

**Table 1:** Five Shu-points & its characteristics.

Five Shu-points (Chinese, Pinyin-English Translation)	Characteristics	Each Yin Primary Meridian	Each Yang Primary Meridian	Total in 12 Primary Meridians
井 Jing-Well	The location where the Meridian Qi emanates	1	1	12

滎 Ying-Spring	The location where the Meridian Qi glides to form a small stream	1	1	12

俞 Shu-Stream	The location where the Original Qi infuses into the Meridian through the function of San Jiao (or Triple Energizer)	1	1	12

原 Yuan-Source	The location where the Original Qi resides and accumulates. In Yin Meridians, the Shu-Stream points are also the Yuan-Source points		1	6

經 Jing-River	The location where the Meridian Qi flows like river	1	1	12

合 He-Sea	The location where the Meridian Qi enters inward and return to the visceral & bowels (*zang-fu*). It is like the confluence of the water into the sea	1	1	12

			Total	66

**Table 2:** Representative Measuring Points used in Ryodoraku.

**RMP Name** **(Hand)**	Meridians	Acupoints	**RMP Name** **(Foot)**	Meridians	Acupoints
**H1**	Lung	LU-9 Taiyuan*∗*	**F1**	Spleen	SP-3 Taibai*∗*
**H2**	Pericardium	PC-7 Daling*∗*	**F2**	Liver	LR-3 Taichong*∗*
**H3**	Heart	HT-7 Shenmen*∗*	**F3**	Kidney	KI-3 Taixi*∗*
**H4**	Small Intestine	SI-4 Wangu*∗*	**F4**	Bladder	BL-65 Shugu
**H5**	San Jiao (Triple Energizer)	TE-4 Yangchi*∗*	**F5**	Gall Bladder	GB-40 Qiuxu*∗*
**H6**	Large Intestine	LI-5 Yangxi	**F6**	Stomach	ST-42 Chongyang*∗*

*∗* indicates Yuan-Source Points. The Acupoints code and name has followed WHO Standard Acupuncture Points Locations [12, 13].

**Table 3:** Major Indices used in the application.

Status	Function	Normal Range	Interpretation
**Physical Status**	It analyzes the average energy balance of all the meridians to evaluate the original Qi (energy) status of the individual.	25-55	Value < 25 indicates Qi deficient while > 55 indicates Qi excessive.

**Metabolism Status**	It analyzes the metabolism status through the assessment of Yin-Yang ratio in the individual.	0.8-1.2	Value < 0.8 indicates high metabolism due to excessive Yang and deficient Yin while > 1.2 indicates low metabolism due to excessive Yin and deficient Yang.

**Musculoskeletal & Circulation Status**	It analyzes the musculoskeletal & circulation status in the individual.	0.8-1.2	Value below or above normal range indicates possible musculoskeletal discomfort such as aches and pain.

**Mental Health Status**	It analyzes the mental health status in the individual.	0.8-1.2	Value below or above normal range indicates possible imbalance in the mental health which one may experience heavy-headed, lightheadedness, insomnia etc.

**Autonomic Nervous System Status**	It analyzes the autonomic nervous system status in the individual.	1.5-2.0	Value below or above normal range indicates underactive or overactive of the autonomic nervous system.

**Table 4:** Baseline assessment.

Parameters	**Male**		**Female**		**P value**
Sample Size	13		30		
	Mean	s.d.	Mean	s.d.	
Age (years)	26.15	6.97	23.27	2.94	0.1725
Body Height (m)	1.70	0.04	1.60	0.06	>0.0001**∗**
Body Weight (kg)	61.8	9.91	54.36	6.38	0.0100**∗**
BMI	21.42	3.10	21.21	2.17	0.8167
Body Temperature (°C)	35.55	0.71	35.38	0.68	0.3342
Left Middle Finger Temperature (°C)	35.81	2.46	33.93	4.43	0.0173**∗**

The values represent the mean difference between the genders.

The statistical significance is indicated with *∗* for p <0.05.

s.d. = standard deviation.

## Data Availability

The data used to support the findings of this study are available from the corresponding author upon request.

## References

[1] Yang W. (1994). Huangdi nei jing ling shu shi jie. *Zhi Yuan Shu Ju*.

[2] Deadman P., Al-Khafaji M., Baker K. (2018). *A Manual of Acupuncture. Point categories*.

[3] Huang W. C. B. (2013). *Zhen jiu ke xue*.

[4] Voll R. (1975). Twenty years of electroacupuncture diagnosis in Germany. A progress report. *AMER.J.ACUPUNCT.*.

[5] Ahn A. C., Martinsen Ø. G. (2007). Electrical characterization of acupuncture points: Technical issues and challenges. *The Journal of Alternative and Complementary Medicine*.

[6] Nakatani Y. (1972). *A Guide for The Application of Ryodoraku Autonomous Nerve Regulatory Therapy*.

[7] Saha A., Walker H. W., Hurst J. W. (1997). The History, Physical, and Laboratory Examinations. *Clinical Methods*.

[8] Boucsein W. (2013). *Electrodermal Activity. “Definitions and Terminology”*.

[9] Critchley H. D. (2002). Electrodermal responses: what happens in the brain. *The Neuroscientist*.

[10] Said G., Krarup C. (2013). Preface. *Peripheral Nerve Disorders*.

[11] Colbert A. P., Yun J., Larsen A., Edinger T., Gregory W. L., Thong T. (2008). Skin impedance measurements for acupuncture research: development of a continuous recording system. *Evidence-Based Complementary and Alternative Medicine*.

[12] WHO Standard Acupuncture Point Locations in the Western Pacific Region. WHO Regional Office for the Western Pacific, 2008

[13] Lim S. (2010). WHO standard acupuncture point locations. *Evidence-Based Complementary and Alternative Medicine*.

[14] "AetoScan Training Handbook," p. 61: AETO TECHNOLOGY CO. LTD: 2018

[15] Tsai M.-Y., Chen S.-Y., Lin C.-C. (2017). Theoretical basis, application, reliability, and sample size estimates of a Meridian Energy Analysis Device for Traditional Chinese Medicine Research. *Clinics*.

[16] Evans W. D., McClagish H., Trudgett C. (1998). Factors affecting the in vivo precision of bioelectrical impedance analysis. *Applied Radiation and Isotopes*.

[17] Sharma B., Hankey A., Nagendra H. R., Meenakshy K. B. (2014). Inter-operator variability of electrodermal measure at Jing Well points using AcuGraph 3. *JAMS Journal of Acupuncture and Meridian Studies*.

[18] Krapohl D., Shaw P. (2015). *Fundamentals of Polygraph Practice*.

[19] Kim H., Richardson C., Roberts J., Gren L., Lyon J. L. (1998). Cold hands, warm heart. *The Lancet*.

[20] Tsai M.-Y., Kuo C.-E., Huang Y.-C., Hsieh C.-L., Chen Y.-H., Chen W.-C. (2014). Meridian energy analysis of the immediate effect of coffee consumption. *European Journal of Integrative Medicine*.

[21] Chamberlin S., Colbert A. P., Larsen A. (2011). Skin Conductance at 24 Source (Yuan) Acupoints in 8637 Patients: Influence of Age, Gender and Time of Day. *JAMS Journal of Acupuncture and Meridian Studies*.

[22] Dahlin M., Joneborg N., Runeson B. (2005). Stress and depression among medical students: A cross-sectional study. *Medical Education*.

[23] Calvarese M. (2015). The Effect of Gender on Stress Factors: An Exploratory Study among University Students. *The Social Science Journal*.

[24] Huang S., Chien L., Chang C., Chen P., Tai C. (2011). Abnormal Gastroscopy Findings Were Related to Lower Meridian Energy. *Evidence-Based Complementary and Alternative Medicine*.

[25] Lin M., Wu H., Hsieh Y. (2012). Evaluation of the Effect of Laser Acupuncture and Cupping with Ryodoraku and Visual Analog Scale on Low Back Pain. *Evidence-Based Complementary and Alternative Medicine*.

[26] Ahn A. C., Schnyer R., Conboy L., Laufer M. R., Wayne P. M. (2009). Electrodermal measures of jing-well points and their clinical relevance in endometriosis-related chronic pelvic pain. *The Journal of Alternative and Complementary Medicine*.

